# Human adipose stem cell differentiation is highly affected by cancer cells both *in vitro* and *in vivo*: implication for autologous fat grafting

**DOI:** 10.1038/cddis.2016.308

**Published:** 2017-01-19

**Authors:** Francesca Paino, Marcella La Noce, Diego Di Nucci, Giovanni Francesco Nicoletti, Rosa Salzillo, Alfredo De Rosa, Giuseppe Andrea Ferraro, Gianpaolo Papaccio, Vincenzo Desiderio, Virginia Tirino

**Affiliations:** 1 Dipartimento di Medicina Sperimentale, Sezione di Biotecnologie, Istologia Medica e Biologia Molecolare, Seconda Università degli Studi di Napoli, Napoli, Italy; 2 Dipartimento di Medicina Sperimentale, Stabulario, Seconda Università degli Studi di Napoli, Napoli, Italy; 3 Dipartimento Multidisciplinare di Specialità Medico-Chirurgiche ed Odontoiatriche, Seconda Università degli Studi di Napoli, Napoli, Italy

## Abstract

Recent studies showed that mesenchymal stem cells derived from adipose tissue can promote tumour progression, raising some concerns regarding their use in regenerative medicine. In this context, we co-cultured either SAOS2 osteosarcoma or MCF7 breast cancer cells with human adipose stem cells (hASCs), in order to evaluate potential effects of cancer cells on hASCs differentiation, *in vitro* and *in vivo*. In this study we observed that both SAOS2 and MCF7 cell lines induced an increase in hASCs proliferation, compared to hASCs alone, but, surprisingly, neither changes in the expression of CD90, CD29, CD324 and vimentin, nor variations in the Twist and Slug mRNAs were detectable. Noteworthy, SAOS2 and MCF7 cells induced in hASCs an upregulation of CD34 expression and stemness genes, including OCT3/4, Nanog, Sox2 and leptin, and a decrease in angiogenic factors, including CD31, PDGF*α*, PDGFR*α*, PDGFR*β* and VEGF. SMAD and pSMAD2/3 increased only in hASCs alone. After 21 days of co-culture, hASCs differentiated both in adipocytes and endothelial cells. Moreover, co-injection of MCF7 cells with hASCs led to the formation of a highly vascularized tumour. Taken together our findings suggest that mesenchymal stem cells, under tumour cell induction, do not differentiate *in vitro* or facilitate the angiogenesis of the tumour *in vivo*, thus opening interesting new scenarios in the relationship between cancer and stem cells. These findings may also lead to greater caution, when managing autologous fat grafts in cancer patients.

Several studies^1-4^ suggested that mesenchymal stem cells (MSCs) participate in tumour development through their homing ability towards the primary tumour and metastatic site, thus playing a pivotal role in tumour progression. One of the physiologic roles of MSCs is being recruited towards the site of wound healing in order to repair injured tissues. The same mechanism is applied to tumours including breast cancer, glioma and osteosarcoma.^5, 6, 7^ In fact, Dvorak^8^ defined cancer as a ‘wound that never heals', stating that the tumour microenvironment is similar to the environment of an injured tissue.

MSCs secrete chemokines, cytokines and growth factors that lead to an inflammatory state, aimed at improving physiological tissue regeneration following injury. However, inflammation also contributes and supports tumorigenesis and metastasis favouring homing of disseminated tumour cells in new tissues.^9^ Cytokines and growth factors mediate a cross-talk between epithelial cells and surrounding stromal cells that are crucial for cancer initiation, progression and metastases formation.^10, 11, 12^ Some factors, such as PDGF and VEGF, released in the tumour microenvironment, can promote tumour neoangiogenesis through the differentiation of endothelial progenitors into new vessels.^13, 14^

Human adipose tissue is a rich source of multipotent MSCs, termed adipose stem cells (hASCs). hASCs are phenotypically similar to bone marrow-MSCs and share multipotentiality, proliferation and the ability to differentiate into mesenchymal lineages. We previously selected hASCs using the CD34 and CD90 markers.^15^ These cells are able to differentiate in multivacuolar adipocytes and endothelial cells forming capillary-like structures in methylcellulose.^15^ In long-term cultures and in murine models, they form vascularized adipose tissue surrounded by connective tissue.^16, 17^ Moreover, hASCs, positive for CD34 and NG_2_ markers and loaded *in vivo* on a cross-linked hyaluronic acid-Lys scaffold, are also able to fabricate skeletal muscle tissue.^18^

Several reports have described that hASCs show regenerative aptitudes in several clinical fields including plastic, orthopaedic, cardiac, bone and breast surgery, promoting tissue repair.^19, 20, 21^

Therefore, hASCs could be promising candidates for reconstructive cellular therapy in patients with cancer history, but the potential risk of promoting tumour reactivation is controversial.

In fact, although hASCs demonstrated good aesthetic results, they could be promoters of cancer recurrence.^22, 23^ Little is known about the underlying molecular mechanisms that link MSCs to tumour cells in the tumour microenvironment. The strict involvement of such interactions has not yet been completely elucidated and some concerns remain regarding the MSCs' potential tumour-suppressive effect or their role in favouring and enhancing tumour development.

In this context, we aimed to ascertain whether mesenchymal and/or epithelial cancer cells may exert any influence upon MSCs derived from adipose tissue. To address this issue, we used a cancer cell line derived from breast cancer, MCF7, and one derived from osteosarcoma, SAOS2, co-culturing both of them with hASCs. In this way, we established a model in which we mimicked the influence of epithelial and/or mesenchymal cancer cells on MSCs microenvironment.

## Results

### MCF7 and SAOS2 cells induced morphologic changes and an increase in hASCs proliferation

**Figure 1:**
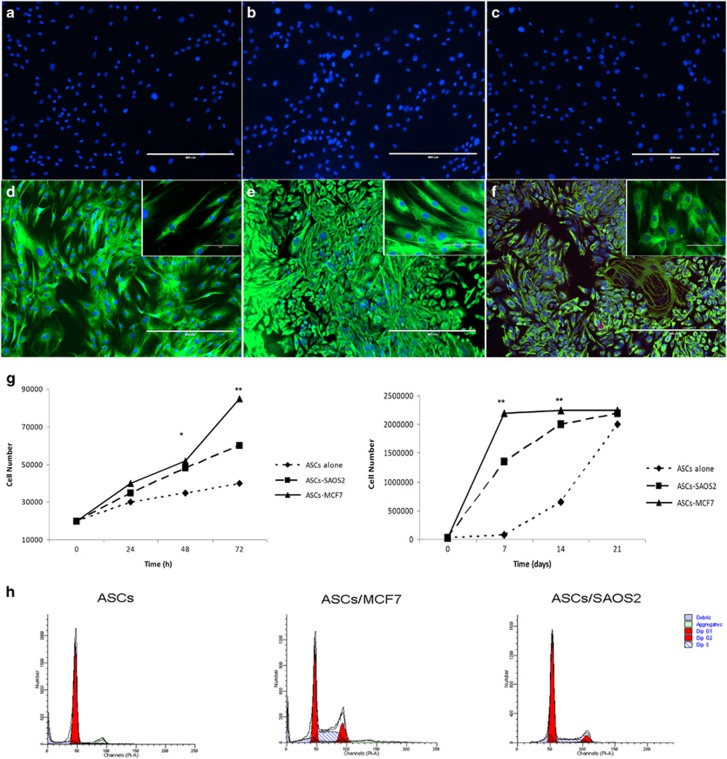
Morphological changes and proliferation in hASCs after cancer cells treatment. (**a–c**) Isotypes controls for immunofluorescence assay on hASCs cultured alone, co-cultured with MCF7 and co-cultured with SAOS2, respectively. (**d**) Vimentin expression on hASCs cultured alone, in the inset, a magnification of hASCs showing typical morphology of fibroblast like cells. (**e**) Vimentin expression on hASCs co-cultured with MCF7 cells, in the inset, a magnification of hASCs showing a mixed morphology of polygonal and elongated cells. (**f**) Vimentin expression on hASCs co-cultured with SAOS2 cells, in the inset, magnification of hASCs showing a morphology of polygonal cells. (**g**) Growth curves at 72 h and 21 days showing proliferation rate of hASCs co-cultured with cancer cells greater than those of hASCs cultured alone. (**h**) Cell cycle analyses showing that hASCs co-cultured with cancer cells are most distributed in S and G_2_M phases. Scale bar=400 μm; inset: scale bar=100 μm. Results are represented as mean±S.E.M. of three independent experiments. *P<0.01; **P<0.001

Independent of culture time, MCF7 and SAOS2 cells in co-cultures led to hASCs morphological alteration. After 3 days of co-culture, MCF7 cells induced the formation of a mixed cell population with elongated and polygonal hASCs cells, as demonstrated by the distribution of vimentin, when compared to hASCs cultured alone (Figures 1a–e). Conversely, SAOS2 cells induced a swelling of hASCs with an epithelioid and/or poligonal shape (Figure 1f), as demonstrated by vimentin distribution. Moreover, hASCs co-cultured with MCF7 cells showed a growth in bundles similar to those of fibroblasts, whereas hASCs co-cultured with SAOS2 cells exhibited a growth in carpet similar to that of epithelial cells (Figures 1e and f).

Regarding proliferation, growth curves, at both short and long time courses were performed. In both cases, SAOS2 and MCF7 cells induced a higher proliferation rate of co-cultured hASCs than hASCs alone. In fact, mean doubling times were 72, 36 and 24 h for hASCs alone, co-cultured with SAOS2 and MCF7, respectively (Figure 1g). Cell cycle analyses also confirmed this result (Figure 1h). hASCs co-cultured with MCF7 and SAOS2 were distributed mainly in S and G_2_M phases with respect to hASCs cultured alone with mean percentages of 38% of cells in S phase and 18% in G_2_M phase for the co-culture with MCF7, and 17% of cells in S phase and 10% in G_2_M phase for the co-culture with SAOS2. hASCs co-cultured alone were mostly distributed in G_0_G_1_ phase with a mean percentage of 92 (Figure 1h).

### MCF7 and SAOS2 cells induced an alteration in CD34 expression in hASCs

**Figure 2:**
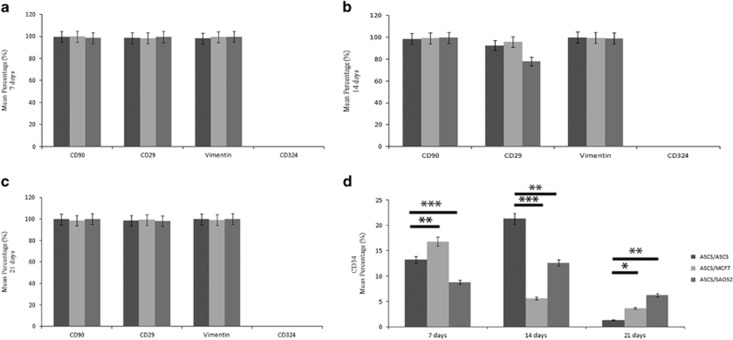
Cytometric analyses of hASCs co-cultured alone and cancer cells. (**a**) CD90, CD29, vimentin, CD324 and CD34 expressions on hASCs at 7. (**b**) 14 and (**c**) 21 days showing no variation of CD90, CD29 and vimentin markers. CD324 expression is negative. (**d**) Cancer cells induce an upregulation of CD34 at 21 days.*P<0.005, **P<0.001, ***P<0.0005 compared to the parental cell line. Results are represented as mean±S.E.M. of three independent experiments

In order to investigate the possible change in the phenotype, cytometric analysis for mesenchymal markers was performed. CD90, CD29 and vimentin expression were similar in all conditions tested with a mean distribution of approximatively 98% (Figures 2a–c). CD34, an epithelial marker and correlated with epithelial to mesenchymal transition, was negative both in hASCs alone and when co-cultured with cancer cells (Figure 2d). Nevertheless, it is noteworthy that a strong variation in CD34 expression was observed (Figure 2d).

At 7 days, MCF7 cells induced an upregulation of CD34 expression compared to both hASCs cultured alone and hASCs co-cultured with SAOS2 cells. At 14 days, they led to a drastic decrease that was stably maintained up to 21 days. On the contrary, at 7 days, SAOS2 cells induced a severe decrease of CD34 expression, an increase at 14 days compared to hASCs cultured alone and a slight decrease at 21 days. At this time, interestingly, albeit the cancer cell-induced decrease, CD34 expression was always higher than that expressed by hASCs cultured alone; furthermore, Saos2 cells led to a greater increase in CD34 expression when compared to the MCF7 cells (Figure 2d).

**Figure 3:**
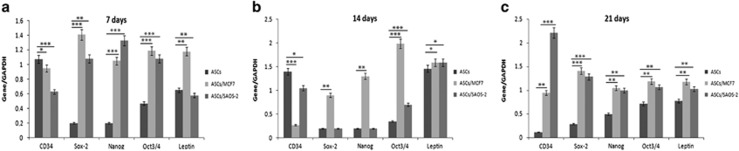
Semiquantitative PCR analyses of stemness markers. (**a**) CD34, Sox2, Nanog, Oct3/4 and leptin mRNA levels on hASCs co-cultured alone and with cancer cells at 7. (**b**) 14 and (**c**) 21 days showing cancer cells induce an increase of stemness factors in hASCs.*P<0.005, **P<0.001, ***P<0.0005 compared to the parental cell line. Results are represented as mean±S.E.M. of three independent experiments

Further investigations of CD34 expression were also performed using RT-PCR (Figure 3). CD34 mRNA levels showed the same trend compared to the one of cytometric analyses. Also in this case, MCF7 cells co-cultured with hASCs induced a strong increase in CD34 mRNA levels at 7 days, a decrease at 14 days and a substantial upregulation at 21 days (Figures 3a–c). For Saos2 cells co-cultured with hASCs, CD34 mRNA levels decreased at 7 days (Figure 3a), increased at 14 days and were always lower than those of hASCs cultured alone (Figure 3b). At 21 days, SAOS2 cells led to a tremendous upregulation of CD34 mRNA levels, compared to those expressed by hASCs cultured alone (Figure 3c). In summary, both MCF7 and SAOS2 induced in hASCs an increase of CD34 marker at both the protein and gene levels.

### MCF7 and SAOS2 cells induced an upregulation of Sox2, Nanog, OCT3/4 and leptin in hASCs

With the purpose of better understanding the effects of cancer cells on hASCs' stemness setting, RT-PCR analyses were performed for stemness genes, including Sox2, Nanog and OCT3/4 (Figure 3). MCF7 cells led to an increase of all stemness genes, Sox2, Nanog and OCT3/4, both during culture and compared to those expressed by hASCs cultured alone. Regarding the effects induced on hASCs, at 7 days, SAOS2 cells induced an upregulation of all three genes of stemness, compared to those expressed by hASCs cultured alone. At 14 days, these genes decreased and no differences were detectable for Sox2 and Nanog between hASCs cultured alone and co-cultured with SAOS2, whereas OCT3/4 decreased with respect to the values found at 7 days, but were found to be still higher than those expressed by hASCs alone. At 21 days, SAOS2 cells surprisingly induced a strong upregulation of Sox2, Nanog and OCT3/4 mRNA levels compared to those of hASCs cultured alone (Figure 3).

Leptin is a growth factor involved specifically in breast tumorigenesis and is produced by hASCs. Interestingly, leptin mRNA levels were increased in hASCs co-cultured both with MCF7 and SAOS2 with respect to those of hASCs cultured alone. Moreover, MCF7 induced a stronger increase of leptin expression than those promoted by SAOS2 cells in hASCs (Figure 3).

In order to evaluate EMT/MET-related genes, TWIST and Slug were analysed by semi-quantitative PCR. No differences were detectable between hASCs cultured alone and those co-cultured with MCF7 and SAOS2 cells (data not shown).

### MCF7 and SAOS2 cells induced a downregulation of angiogenic factors in hASCs

**Figure 4:**
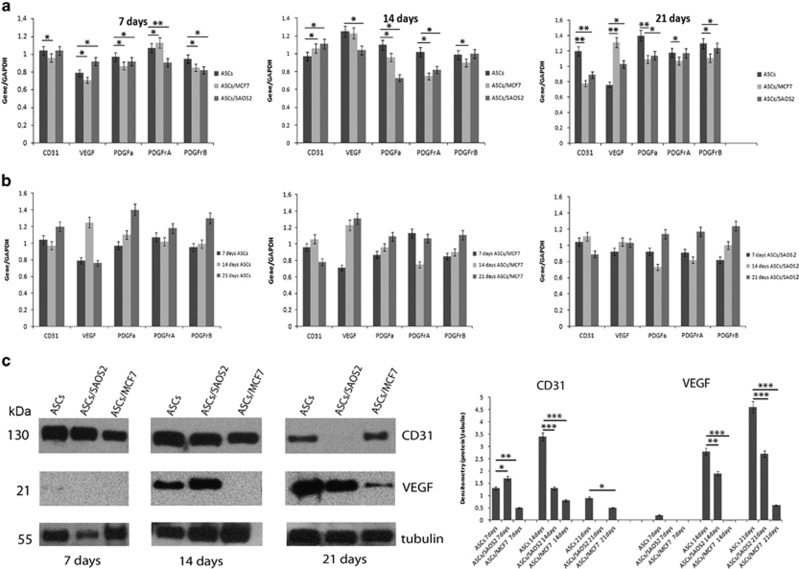
Semiquantitative PCR and western blot analyses of endothelial markers**. (a**) Variation of CD31, VEGF, PDGFA, PDGFR*α*, PDGFR*β* mRNA levels at 7, 14 and 21 days showing cancer cells induce a downregulation of CD31, PDGFA, PDGFR*α*, PDGFR*β* gene expression, and an upregulation VEGF levels in hASCs. (**b**) Variation of CD31, VEGF, PDGFA, PDGFR*α*, PDGFR*β* mRNA levels in culture time showing endothelial gene expression increases in hASCs alone. (**c**) Variation of CD31 and VEGF protein levels at 7, 14 and 21 days showing a decrease of these markers in hASCs induced by cancer cells. The protein amount was normalized to *β*-tubulin and evaluated using densitometry histogram. *P<0.005, **P<0.001, ***P<0.0005 compared to the parental cell line. Results are represented as mean±S.E.M. of three independent experiments

With the aim of evaluating the effects of cancer cells upon the hASCs' capability of promoting angiogenesis, CD31, VEGF, PDGFA, PDGFR*α*, PDGFR*β* gene expression was analysed at 7, 14 and 21 days (Figure 4). At 7 days, MCF7 cells induced a downregulation of every angiogenic factor except for PDGFR*β* in hASCs, when compared to those of hASCs cultured alone. At 14 days, only an increase in the CD31 mRNA level was detected. VEGF mRNA levels remained similar for hASCs co-cultured with MCF7 and hASCs cultured alone. PDGFA, PDGFR*α*, PDGFR*β* genes showed a decrease. At 21 days, MCF7 led to a downregulation of all markers except for VEGF mRNA level compared to those of hASCs cultured alone (Figure 4a). On the other hand, SAOS2 cells induced in hASCs an increase in only VEGF mRNA levels at 7 days, an increase in CD31 mRNA levels at 14 days, and a strong decrease in all angiogenic factors except for VEGF mRNA levels at 21 days compared to those of hASCs cultured alone (Figure 4a).

In addition, during culture, hASCs cultured alone showed a strong increase in angiogenic factors indicating their differentiation versus the angiogenic lineage. Also in hASCs co-cultured with MCF7 and SAOS2 cells, it was possible to observe a similar trend, but with a slower increase in angiogenic genes compared to those observed in hASCs cultured alone (Figure 4b).

To further characterize angiogenic differentiation, expression of CD31 and VEGF proteins were analysed at 7, 14 and 21 days by western blotting. In accordance with gene expression analyses, cancer cells induced a decrease both of CD31 and VEGF in hASCs in comparison to those of hASCs cultured alone, reinforcing the hypothesis of angiogenic inhibition induced by cancer cells. In particular, at 7 days, SAOS2 induced an increase of CD31 expression respect to those of hASCs cultured alone, whereas at 14 and 21 days, CD31 expression decreased. MCF7 induced a strong decrease at all-time points in comparison to those of hASCs cultured alone. For VEGF expression at 7 days, only hASCs, cultured alone, showed a weak expression of this marker. At 14 and 21 days, both SAOS2 and MCF7 cells induced a drastic decrease of VEGF protein compared to those of hASCs cultured alone (Figure 4c).

### Adipogenic and angiogenic differentiation evaluation

**Figure 5:**
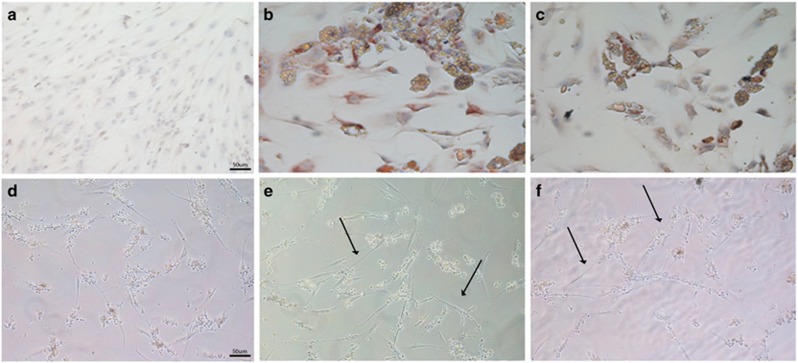
Evaluation of hASCs differentiation ability by immunohistochemical analysis**. (a**) Negative expression of adiponectin on hASCs. (**b**) Strong positivity of adiponectin on hASCs co-cultured with MCF7. (**c**) Positivity for adiponectin on hASCs co-cultured with SAOS2. (**d**) hASCs alone are not able to form a vascular network in matrigel medium; (**e**) hASCs co-cultured with MCF7 formed intercellular tubes network with endothelial morphology (arrows). (**f**) hASCs co-cultured with SAOS2 formed structures similar to capillary like tubes (arrows). Scale bar=50 *μ*m

After 21 days of co-culture, hASCs were tested for their ability to differentiate using an adipogenic medium for adipogenic differentiation and matrigel for angiogenic differentiation for 7 and 15 days, respectively (Figure 5). hASCs cultured alone were not able to form multivacuolar adipocytes. The cells were negative for adiponectin and no formation of intracellular lipid droplets was detectable. They showed an elongated shape, similar to fibroblasts (Figure 5a). On the contrary, hASCs co-cultured with both MCF7 and SAOS2 cells, acquired a typical morphology of adipocytes with lipid droplets, positive for adiponectin, confirming adipogenic differentiation (Figures 5b and c). For the angiogenic differentiation, hASCs alone were not able to form a vascular network. The cells were spread on matrigel without acquiring any morphological changes in structures that were like vessels (Figure 5d). Conversely, hASCs co-cultured with MCF7 and SAOS2 cells formed a network of intercellular tubes including cells with endothelial morphology, thus confirming their angiogenic differentiation (Figures 5e and f).

### MCF7 and SAOS2 induced a downregulation of p-SMAD2/3 in hASCs

**Figure 6:**
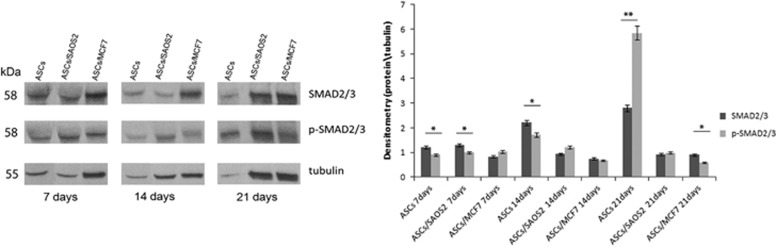
Western blot analysis and densitometry evaluation of SMAD2/3 and p-SMAD2/3. pSMAD2/3 was activated only in hASCs cultured alone. The protein amount was normalized to tubulin. *P<0.005, **P<0.001, compared to the parental cell line. Results are represented as mean±S.E.M. of three independent experiments

In order to investigate the effect of cancer cells on SMAD2/3 and its phosphorylated form, a western blot assay was performed on hASCs cultured alone and with MCF7 and SAOS2 at 7, 14 and 21 days (Figure 6). hASCs cultured alone showed a strong increase of both SMAD2/3 and p-SMAD2/3 over the culture period and p-SMAD2/3 levels increased dramatically at 21 days. hASCs co-cultured with MCF7 and SAOS2 cells always expressed lower levels of both SMAD2/3 and p-SMAD2/3 compared to hASCs cultured alone. In particular, MCF7 cells induced a decrease in p-SMAD2/3 during culture time when co-cultured with hASCs, whereas SAOS2 cells led to a peak of p-SMAD2/3 at 14 days followed by a decrease at 21 days in co-cultured hASCs (Figure 6). In summary, p-SMAD2/3 was activated only in hASCs cultured alone.

### Co-injection of MCF7 and hASCs promoted tumour formation in xenografts

**Figure 7:**
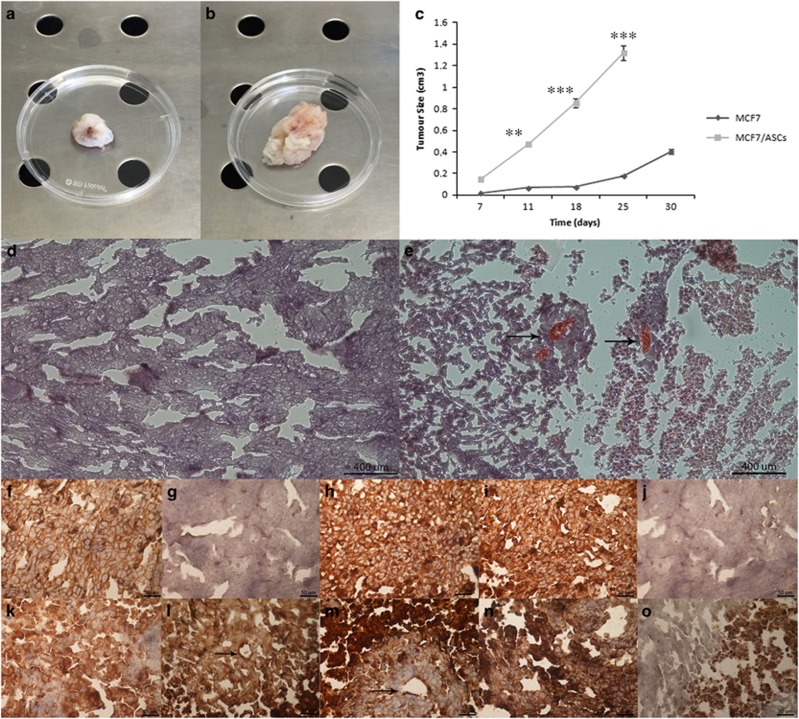
Xenograft evaluation. (**a, b**) Xenografted tumour derived from implantation of MCF7 alone and MCF7 with hASCs. (**c**) Xenografted tumour growth curves show that the injection of MCF7 with hASCs form tumours with major efficiency in comparison with injection of MCF7 alone. (**d, e**) H&E staining of xenografted tumour derived from implantation of MCF7 alone and MCF7 with hASCs. Scale bar=400 μm. (**f-j**) Positivity for I Class HLA, negativity for CD31, positivity for VEGF and cytokeratin, negativity for vimentin on xenografted tumour derived from implantation of MCF7 alone. (**k-o**) Positivity for I Class HLA, CD31, VEGF, cytokeratin and vimentin on xenografted tumour sample derived from implantation of MCF7 with hASCs. Arrows indicate blood vessels. Scale bar=50 *μ*m. **P<0.001, ***P<0.0005 compared to the parental cell line. Results are represented as mean±S.E.M. of three independent experiments

MSCs home into the human tissue stroma for tissue regeneration. To evaluate whether the co-injection of MCF7 cells with hASCs could influence the behaviour of MCF7 breast cancer cells, *in vivo* tumorigenicity was analysed. hASCs were not tumorigenic *per se*; however, they revealed a tumorigenic potential in the presence of MCF7 cells. In fact, both MCF7 cells and MCF7 co-injected with hASCs formed tumours in mice after 30 days. Moreover, although both MCF7 cells alone and MCF7 co-injected hASCs had the capability to regenerate tumours, the ratio of tumour size and growth of MCF7 with hASCs cells were significantly greater than those of MCF7 cells alone (Figures 7a–c). MCF7 cells with hASCs gave rise to 10-fold greater tumour sizes than those detected in MCF7 cells alone (Figure 7c). Hematoxylin and eosin (H&E) staining revealed that xenografted tumours consistently reproduced the original human mammary tumour (Figures 7d and e), as confirmed also by positive staining for class I HLA (Figure 7f). Moreover, xenograft tumours derived from the co-injection of MCF7 with hASCs were highly vascularized. Xenografted tumours arose from the injection of MCF7 cells alone showed positivity for class I HLA, thus confirming human origin, negativity for CD31, positivity for VEGF and cytokeratin, and negativity for vimentin (Figures 7f–j). On the other hand, xenografts derived from co-injection of MCF7 cells with hASCs exhibited positivity for class I HLA, CD31, VEGF and slightly positive for vimentin and cytokeratin (Figures 7k–o).

## Discussion

MSCs derived from adipose tissue represent a promising cell source for regenerative therapies. Recent studies demonstrated that hASCs can improve graft retention.^24, 25^ However, the risks linked to cell treatment still remain unclear, particularly in the context of patients affected by pre-existing cancer.^26, 27^ MSCs derived from adipose tissue could serve as a primary MSC source for cancer contributing to the tumour microenvironment and thus playing a role both in epithelial and mesenchymal carcinogenesis.^28^ Therefore, it is likely that adipose tissues adjacent to the tumour may be a more significant contributor of hASCs for cancer progression. All studies of literature have shown, up to now, the effects of MSCs on cancer cells.^2, 5, 17, 18, 19, 20, 21^ Our aim was, on the contrary, to investigate the effects that cancer cells of both epithelial and mesenchymal origin may exert on hASCs still present after tumour resection, a not uncommon scenario. To address this issue, we used MCF7 cells in order to mimic the tumour microenvironment of mammary glands surrounded by adipose tissue with its resident adipose stem cells and SAOS2 cells to mimic the stromal tissue sustaining mesenchymal tumours. We established a co-culture system using inserts of 0.4 μm. We found that both MCF7 and SAOS2 stimulated hASCs increasing their proliferation. In fact they showed an almost halved doubling time, being distributed in S-G_2_M phase of the cell cycle, compared to hASCs cultured alone. Thus, the first effect of tumour cells on stem cells was to increase their proliferation. One hypothesis is that cancer cells could induce such an effect with the purpose to increase the number of stem cells that will then be beneficial to the tumour itself in order to favour specific processes ranging from growth of vessels to metastases. Generally, when stem cells proliferate, they differentiate in a specific lineage. In previous studies,^15, 16, 17^ we demonstrated that hASCs differentiate in endothelial cells without inducing factors. Therefore, we investigated a series of markers involved in stemness maintenance, EMT/MrET processes and differentiation. No changes were detectable for mesenchymal markers except for the CD34 marker.

CD34 is a master marker by which hASCs are isolated from the vascular stromal fraction of adipose tissue.^15, 16, 17^ They are expressed by hematopoietic stem cells and endothelial and mesenchymal cells of different healthy tissues.^29, 30^ The expression of CD34 has been extensively studied in the reactive stroma of many types of cancer, particularly in breast cancer.^31, 32^ In normal tissues, CD34^+^ fibroblasts are predominantly located in the region surrounding small vessels adjacent to the basal lamina of the epithelial layer.^33^ Some authors suggested that CD34^+^ fibroblasts in breast and sarcoma lesions are recruited through the bloodstream.^32, 34^ In general, hASCs show an increase in CD34 expression and then a decrease during differentiation.^15^ This biological behaviour was not observed for hASCs co-cultured with cancer cells in that an increase in the induction of CD34 was observed. Another interesting consideration is that stemness factors including NANOG, OCT3/4 and Sox2 increased in concordance with CD34 during cell culture. Cancer cells, both MCF7 and SAOS2, induced both an upregulation and the maintenance of stemness of hASCs during culture. This important result was confirmed by a decrease of p-SMAD2/3. Singh and colleagues^35^showed that when the phosphorylation level of SMAD2/3 decreased, the level of Nanog increased, and consequently the differentiation decreased. The induction and maintenance of a stem phenotype are also confirmed by the evidence that the mesenchymal-epithelial transition (MET) is not induced. In our study, MET-related markers including CD324, Slug, Twist and vimentin did not change after cancer cell treatment.

Therefore, we suppose that cancer cells, independent from their origin, induce the maintenance of stemness and stability of hASCs in the microenvironment in which they live without the activation of migration signalling. This is of paramount importance because for the cancer cell it is easier to use a stem cell, resident in its microenvironment, than to recruit it from bone marrow. The hASCs' stemness phenotype was confirmed also by the endothelial/angiogenic gene expression analyses, as well as by CD31 and VEGF proteins analyses, which clearly showed that these factors were upregulated in hASCs cultured alone and strongly downregulated in hASCs co-cultured with cancer cells, thereby reinforcing the hypothesis of a ‘stemness steady state' or maintenance. Another stemness feature is linked to the ability to differentiate. hASCs, after co-culture with cancer cells, were able to differentiate into adipocytes and endothelial cells. hASCs cultured alone were not able to form adipocyte and capillary-like structures.

Additionally, in order to understand the role of hASCs in tumour growth, we performed a tumorigenesis assay. We used only MCF7 cells and not SAOS2 cells because it is well known that these cells do not grow in murine models. Co-injection of MCF7 cells and hASCs into nude mice suggested that hASCs did not differentiate in adipocytes, but they were integrated into the tumour stroma subsequently forming tumours that grew faster and bigger than those originating from MCF7 cells alone, thus reinforcing the hypothesis that hASCs have the capability of sustaining tumour growth. Furthermore, tumours derived from co-injection of MCF7 and ASCs were vascularized being VEGF and CD31 highly expressed. Our hypothesis, therefore, is that cancer cells of both epithelial and mesenchymal origin release factors to induce and maintain a stem cell phenotype in hASCs, which modify their programme of differentiation with the purpose of assisting the tumour in its development, contributing to the formation of new blood vessels. The creation of a stem microenvironment surrounding the tumour mass appears to be a possible mechanism to explain the survival and growth of the tumour. Cancer cells and stem cells share several biological properties, and transcriptional factors that monitor the fate of stem cells could play a major role in the renewal of cells modified from the cancerous microenvironment. Therefore, the induction and maintenance of a stemness phenotype in mesenchymal cells might be a further mechanism of survival and resistance to drugs implemented by the tumour. The identification of factors secreted by cancer cells that induce such changes or specific markers that characterize the new modified cells could be useful in strengthening the conventional treatments and combat the relapse of the disease.

In conclusion, our study addresses for the first time that cancer cells are able to maintain hASCs in a ‘stemness state'. Thus, if cancer cells persist following surgery, they will most likely induce resident hASCs to promote tumour angiogenesis, thus exacerbating tumour growth and aggressiveness. Consequently, adipose grafts may give rise, in the case of cancer cell persistence after surgery (a rather common event), to tumour growth. Therefore, it must be strongly discouraged in groups of patients including those undergoing:adipose graft after a breast cancer for mastoplasty; adipose graft, following cancer in general for every treatment.

In these circumstances, the use of adipose tissue for auto-grafting must be carefully performed, only after meticulous analyses of possible cancer. Also the cases of an early cancer, which is made of only a few cells, though not yet detectable, must be regarded as a possible side effect or contro-indication and the informed consent must include this prospect.

This work highlights the biological elucidation for those clinical cases reporting a fast-growing and more aggressive recurrence subsequent to fat grafting in patients with a history of cancer.

## Materials and methods

### Ethics statement

All animal experiments were conducted in full compliance with Second University of Naples and Italian Legislation for Animal Care.

### Cell culture

SAOS2 and MCF7 cell lines were purchased from ATCC cell bank; cells were placed in DMEM culture medium (Gibco, Rodano, Milan, Italy), supplemented with 10% FBS (Gibco, Rodano, Milan, Italy), 100 mM 2 P-ascorbic acid, 2 mM L-glutamine, 100 U/ml penicillin, 100 mg/ml streptomycin (all purchased from Invitrogen, Life Technologies Italia, Monza, Italy) and placed in 75 ml flasks with filtered valves. Flasks were incubated at 37 °C in 5% CO_2_ and the medium was changed twice a week. At confluence, cells were subdivided into new flasks until the end of the experiment.

### Adipose tissue collection and cell culture

Subcutaneous adipose tissue from abdomen and breast was obtained following written informed consent, approved by our Internal Ethical Committee (Second University Ethical Committee), from patients with a mean age of 37±2.5 years. Adipose tissue was obtained through lipectomy or liposuction in the Plastic and Reconstructive Surgery Division of the Second University of Naples. The adipose tissue was placed in a physiological solution (0.9% NaCl), washed twice in PBS, minced, and placed in a digestion solution: collagenase type I (3 mg/ml; Gibco, Rodano, Milan, Italy) and dispase (4 mg/ml; Gibco, Rodano, Milan, Italy) at 37 °C for 60 min in a shaking water bath. The digest was filtered through 70 μm filters (Becton & Dickinson, Sunnyvale, CA, USA). After filtration and washing, the pellet was re-suspended in erythrocyte lysis buffer (155 mM NH_4_Cl, 10 mM KHCO_3_, 0.1 mM EDTA, pH 7.3) for 10 min at room temperature. The cell suspension was centrifuged at 1300 rpm for 7 min and the pellet re-suspended in DMEM at 10% FBS in 25 cm^2^ flasks. hASCs were isolated and characterized by immunophenotype as previously described in De Francesco *et al.*^15^ hASCs were used at first passage of culture. Flasks were incubated at 37 °C under 5% CO_2_ and the medium changed twice a week. Cells reached confluence in 5–7 days.

### Co-cultures

hASCs and SAOS2 or MCF7 cells were plated in co-culture in equal numbers with a 0.4 μm insert (Nunc, Thermo-Fisher Scientific, Milan, Italy) in DMEM at 10% FBS. hASCs were plated at 1° passage of culture, 10 000 cell/well in quadruplicates. hASCs in standard culture condition were used as control cell lines.

### Growth and cell cycle analyses

hASCs alone and co-cultured with SAOS2 and MCF7 cells were plated at a density of 20 × 10^4^ cells/well in six-well plates. At 24, 48 and 72 h and 7, 14 and 21 days, cells were harvested and re-suspended in PBS. An aliquot of cell suspension was diluted with 0.4% trypan blue (Sigma-Aldrich, Milan, Italy), pipetted onto a haemocytometer and counted under a microscope at × 200 magnification. Live cells excluded the dye, whereas dead cells take up the dye and consequently stained intensely with trypan blue. The number of viable cells for each experimental condition were counted and represented on a linear graph. The doubling time (DT) was determined from the growth curves or by using the formula:


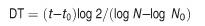


where *t* and *t*_0_ were the times at which the cells were counted, and *N* and *N*_0_ were the cell numbers at times *t* and *t*_0_, respectively. Experiments were repeated three times with three triplicates for each experiment.

Cell cycle anlysis assay was performed using flow cytometry. After 72 h of co-culture, hASCs, cultured alone and with MCF7 and SAOS2 cells, were harvested in PBS containing 2 mM EDTA, washed once with PBS, fixed in iced ethanol 70% and incubated with 25 μg/ml PI (Sigma-Aldrich) plus Rnasi 1 mg/ml for 120 min at 4 °C in the dark. Stained nuclei were analysed with a FACS Aria III (Becton & Dickinson, Mountain View, CA, USA), and the data analysed using a ModFit 2.0 cell cycle analysis software (Verity Software House, Topsham, UK). Experiments were repeated three times with three triplicates for each experiment.

### Flow cytometry

Following isolation, the cells were expanded *in vitro* and then characterized with flow cytometry in order to evaluate the cell surface marker expression at 7, 14 and 21 days of co-culture both with SAOS2 and MCF7 cells. hASCs were characterized with antibodies against the following markers: CD90 FITC, CD34 PE, CD29 PECy5.5, CD44 FITC, CD324 PE and vimentin (from BD Pharmingen, Milan, Italy). For staining, the antibodies were incubated for 30 min at 4 °C. After washing, cells were re-suspended in PBS. For intracellular staining of vimentin, Fix & Perm kit (Invitrogen, Life Technologies Italia) was used following the manufacturer's instructions. All samples were analysed using Diva Software for flow cytometer FACS ARIA III (BD Pharmingen). Statistical evaluation was obtained from three independent experiments. Standard deviation was indicated by error bars.

### Immunofluorescence

hASCs cultured alone and co-cultured with SAOS2 and MCF7 cells were fixed with 4% paraformaldehyde, permeabilized with TRITON X-100 and blocked with bovine serum albumin at 5% for 1 h at room temperature and then stained with primary antibodies at 4 °C overnight. The primary antibody used was mouse anti-human vimentin (Invitrogen, Life Technologies Italia). The secondary antibody, goat anti-mouse FITC (Abcam, Cambridge, UK) diluted 1:200 in PBS, was incubated for 60 min at 4 °C, and the Hoechst33342 (Invitrogen, Life Technologies Italia) was used to stain the nucleus and was incubated for 7 min at room temperature. Cells were observed under the fluorescence microscope (EVOS, Life Technologies, Milan, Italy). Isotypes and non-probed cells were used as controls.

### Adipogenic differentiation

After 21 days of co-culture with MCF7 and SAOS2 cells, hASCs were induced in the following adipogenic medium for 2–3 weeks: DMEM supplemented with 10% FBS plus dexamethasone (1 mM; Sigma-Aldrich), human recombinant insulin (10 mM; Sigma-Aldrich), indomethacin (200 mM; Fluka, Milan, Italy) and 3-isobutyl-1-methyl-xantine (IBMX) (0.5 mM; Sigma-Aldrich). Cells cultured in basal medium were used as controls. To confirm the adipogenic differentiation, cells were fixed with 4% paraformaldehyde (Sigma-Aldrich) for 10 min at 4 °C, washed in PBS and stained with primary antibody mouse anti-human adiponectin (Abcam) diluted 1:100 in PBS for 30 min at 4 °C using an Abcam Kit (Abcam), according to the manufacturer's instructions. The nuclei were stained with haematoxylin and the cells were observed under an inverted light microscope.

### Angiogenic differentiation

After 21 days of co-culture with MCF7 and SAOS2 cells, to analyse *in vitro* capillary-like morphology, hASCs were plated in 24-well plates in a semi-solid growth medium that consisted of matrigel in DMEM, 20% FBS, 1% bovine serum albumin, 1024 mol/L mercaptoethanol (Sigma-Aldrich) and 2 mmol/L L-glutammine (Gibco, Rodano, Milan, Italy). All cultures were performed in triplicate, incubated at 37 °C under 5% CO_2_ and left for 7 days to develop a capillary-like morphology.

### RNA extraction and RT-PCR

Total RNA was extracted from ASCs after 7, 14 and 21 days of culture in DMEM supplemented with 10% FBS, 10% FBS New Zealand or 10% HS, using an AMBION kit (Life Technologies Italia) following the manufacturer's instructions. RNA was treated with DNase (Promega, Milan, Italy) to exclude DNA contamination and stored at −80 °C until required. cDNA synthesis was carried out from total RNA (1 μg) using VILO SUPERSCRIPT (Invitrogen, Life Technologies Italia). PCR analyses were carried out using a BIOER Life Pro thermal cycler (Life Technologies Italia) in which samples underwent a 2-min denaturing step at 94 °C, followed by 35 cycles of 94 °C for 30 s, 52–60 °C for 60 s, 72 °C for 30 s, and a final extension step at 72 °C for 4 min. Each PCR reaction was performed in a total volume of 12.5 μl containing Tris buffer 10 mM pH 8, 0.2 mM of each dNTP, 1.5 mM MgCl_2_, and 0.2 μM of each primer, Taq DNA polymerase 1 U and 1 μl of each cDNA. PCR was performed using the following primer sequences and PCR product annealing: GAPDH, fw: GGAGTCAACGGATTTGGTCG, rev: CTTCCCGTTCTCAGCCTTGA, 57 °C; CD34, fw: TCAAATGTTCAGGCATCAGAG, rev: TCAGGTCAGATTGGTGCTT, 56 °C; NANOG, fw: TTCAGTCTGGACACTGGCTG, rev: CTCGGTGATTAGGGTCCAAC, 58 °C; SOX-2, fw: CGATGCCGACAAGAAAACTT, rev: CAAACTTCCTGCAAAGCTCC, 58 °C; OCT3/4, fw: ACATGTGTAAGCTGCGGCC, rev: GTTGTGCATAGTCGCTGCTTG, 58 °C;VEGF, fw: TGACAGGGAAGAGGAGGAGA, rev: CGTCTGACCTGGGGTAGAGA, 59 °C; PDGFA, fw: ACACGAGCAGTGTCAAGTGC, rev: GGCTCATCCTCACCTCACAT, 60 °C; PDGFR*α*, fw: GAAGCTGTCAACCTGCATGA, rev: CTTCCTTAGCACGGATCAGC, 57 °C; PDGFR*β*, fw: GCACTTTTATCCACCCAGGA, rev: GTACTTGGCTCAGCCTCCAG, 60 °C; CD31, fw: ATTGCAGTGGTTATCATCGGAGTG, rev: CTCGTTGTTGGAGTTCAGAAGTGG, 58 °C; LEPTIN, fw: AAGCTTCAGGCTACTCCACA, rev: TGGAAGAGTGGCTTAGAGGA, 58 °C; SLUG, fw: GAGCATTTGCAGACAGGTCA, rev: CCTCATGTTTGTGCAGGAGA, 58 °C; TWIST, fw: TCTCGGTCTGGAGGATGGAG; rev: GTTATCCAGCTCCAGAGTCT, 58 °C. The amplification products were separated on a 2% agarose gel in Tris-acetate EDTA (TAE) buffer. The transcript amount of each gene was normalized to GAPDH. Relative expression was calculated using Image J.

### Western blot assay

For western blot analyses, cells were lysed in RIPA buffer (50 mM Tris-HCl pH 7.2, 150 mM NaCl, 1% NP40, 0.1% SDS, 0.5% DOC, 1 mM PMSF, 25 mM MgCl2, and supplemented with a phosphatase inhibitor cocktail). Protein concentration was determined by the BCA assay (Bio-Rad Laboratories, Hercules, CA). Equivalent amounts of protein (50 μg) were electrophoresed on 10% SDS–polyacrylamide gels. Precision Plus Protein™ Dual Color Standards (Bio-Rad) were used to determine molecular weight. The gel was electroblotted onto nitrocellulose membrane by using a Trans Blot Turbo system (Bio-Rad) following the manufacturer's instructions. The membrane was blocked with 5% milk in TBS-0.1% Tween (TTBS) for 1 h at RT and washed with TTBS. The membrane was then incubated with specific primary anti-human antibodies against Smad-2/3 (1:1000; BD Pharmingen), p-Smad-2/3 (1:1000; Invitrogen, Life Technologies Italia), CD31 (1:1000; Abcam), VEGF (1:1000; Santa Cruz Biotechnology, Inc., Heidelberg, Germany) or *β*-Tubulin (1:5000; Abcam) overnight at 4 °C. The membrane was then washed with TTBS and incubated with the appropriate HRP-conjugated secondary antibody diluted 1:5000 in 3% milk in TTBS for 1 h at RT. Membrane was then washed three times with TTBS. Immunoreactive protein bands were visualized by the Pierce™ ECL Western Blotting Substrate (Thermo Scientific, Milan, Italy) according to the manufacturer's instructions. The protein amount was normalized to tubulin. Relative expression was calculated using Image J. Experiments were repeated three times with three triplicates for each experiment.

### *In vivo* transplantation of cancer cells and hASCs

For *in vivo* experiments, hASCs alone or MCF7 cells with hASCs were subcutaneously injected in Balb/c nude mice. Mice were purchased from Charles River (Charles River Laboratories International, Inc, Milan, Italy) and acclimatised for a week prior to experimentation.

Cancer cells and ASCs were enzymatically dissociated to obtain single-cell suspensions, suspended in 0.2 ml of PBS, and injected subcutaneously in the right flank of 6-week-old female Balb/c nude mice at a density of 1 × 10^6^ cells. PBS alone was administered in the left flank of mice as a control. Ten mice for group were used. The day of injection was considered day 0. Xenograft tumours were measured and mice were weighed once a week. After 30 days, mice were killed and tissues were collected, fixed in buffered formalin and subsequently analysed by immunohistochemistry.

Tumour volume was determined by callipers with the following formula: (L × W^2^)/2 =  mm^3^ where L and W are the longest and shortest perpendicular measurements in millimeters, respectively. Tumour growth data were derived from three independent experiments.

Hematoxylin and eosin (H&E) staining and immunohistochemical analyses for Class I HLA, VEGF, CD31, vimentin and cytokeratin (all purchased from Abcam) were performed to determine tumour histology and phenotype, using an Abcam Kit (Abcam) and according to the manufacturer's instructions. The injection experiments were made in triplicate. This examination was performed according to our internal ethics committee.

### Statistical analysis

Experiments were performed in quadruplicates. Student's *t*-test (two-tailed) was used for statistical evaluation. Data from at least three independent experiments are represented as mean±S.E.M. Level of significance was set at *P*<0.05.
